# Retinal Nerve Fibre Layer Thickness Change Following Femtosecond Laser-Assisted *in situ* Keratomileusis

**DOI:** 10.3389/fmed.2021.778666

**Published:** 2021-11-29

**Authors:** Yang Jiang, Zhonghai Wang, Ying Li, Yong Li, Thomas Chengxuan Lu

**Affiliations:** ^1^Department of Ophthalmology, Peking Union Medical College Hospital, Chinese Academy of Medical Sciences, Beijing, China; ^2^Key Laboratory of Ocular Fundus Diseases, Chinese Academy of Medical Sciences, Peking Union Medical College, Beijing, China; ^3^Department of Ophthalmology, Xi'an Fourth Hospital, Xi'an, China; ^4^School of Medicine, University of New South Wales, Kensington, NSW, Australia

**Keywords:** femtosecond laser-assisted *in situ* keratomileusis, macular thickness, retinal nerve fibre layer thickness, optical coherence tomography, myopia

## Abstract

**Purpose:** To evaluate the effect of femtosecond laser-assisted *in situ* keratomileusis (FS-LASIK) on retinal fovea thickness, volume, and retinal nerve fibre layer (RNFL) thickness.

**Methods:** Thirty-seven eyes (37 patients) undergoing FS-LASIK were included in this prospective study. Optical coherence tomography (OCT) was performed 1 day before, 1 h and 1 day after FS-LASIK surgery.

**Result:** Eighteen male and nineteen females were enrolled. Mean patient age was 22.94 ± 4.22 years. One hour postoperatively, macula fovea thicknesses, macula fovea volume, macula parafovea thickness, macula parafovea volume, macula perifovea thickness, macula perifove volume, temporal RNFL thickness, and superior RNFL thickness measures showed significant decrease (*t* = 6.171, 6.032, and 9.837, 9.700, 2.532, 4.393, 4.926, 2.265; *p* = 0.000, 0.000, 0.000, 0.000, 0.016, 0.000, 0.000, and 0.011). Day 1 post-operation, macula fovea thicknesses, macula fovea volume, macula parafovea thickness, macula parafovea volume, and inferior RNFL thickness measures showed significant change compared to preoperative measures (*t* = 3.620, 3.220, 2.901, 2.910, 3.632; *p* = 0.001, 0.003, 0.006, 0.006, and 0.001).

**Conclusion:** Our data suggest there are alterations in retinal foveal and RNFL measurements by OCT 1 h and 1 day after FS-LASIK surgery.

## Introduction

Laser-assisted *in situ* keratomileusis is a popular corneal refractive technique utilised to enhance visual acuity. Both traditional microkeratome and modern femtosecond laser laser-assisted *in situ* keratomileusis (FS-LASIK) involve the dissection of a superficial lamellar flap by suction to reveal the corneal stroma for remodelling. FS-LASIK creates a predictable homogeneously thick stromal flap, which is elevated by a suction ring. It leads to better refractive results in comparison to standard microkeratomes most likely due to more predictable and planar corneal flaps (1).

During the LASIK procedure, the intraocular pressure (IOP) is abruptly increased. Dramatic IOP change has been theorised to vitreous traction, thereby causing postoperative optic nerve and vitreoretinal complications. As such, glaucoma is also a contraindication (2, 3). It is evident from the literature that LASIK with mechanical microkeratome is not detrimental to retinal neve fibre layers of healthy individuals (4, 5). The evidence surrounding FS-LASIK and foveal and retinal thickness is conflicting and sparse. Some studies have demonstrated changes within the retina, following FS-LASIKS (6), whilst other have not (7), and there is uncertainty in the clinical significance of such change (8).

The objective of this study was to determine whether FS-LASIK induces changes in retinal foveal and RNFL measurements with optical coherence tomography (OCT) immediately post-procedure.

## Materials and Methods

All patients underwent corneal pachymetry and topography preoperatively. Patients with contraindication to LASIKs were excluded. Contraindications include ocular disease, ocular surface disorder, glaucoma, corneal thickness <500 microns, and/or irregular corneal topography. The patients were also excluded from the study if their preoperative best-corrected visual acuity was <20/40 or if they had prior laser or intraocular surgery. Our prospective study recruited patients undergoing FS-LASIK by one surgeon (WZH).

All patients on examination had an intraocular pressure (IOP) <21 mmHg and normal optic disk. A normal optic disc was defined by a vertical cup-to-disk asymmetry <0.2, cup/disk ratio <0.6, and an intact neuro-retinal rim without peripapillary haemorrhages, notches, localised pallor, or nerve fibre layer defect.

All patients underwent FS-LASIK treatment using the VISX™ (Abbott Medical Optics Inc., Santa Clara, CA, USA) under topical anaesthesia. A corneal flap, 110-micron thick, was created by IFS IntraLase™ 150 HZ (Abbott Medical Optics Inc., Santa Ana, CA, USA). Suction during the creation of a flap lasted ~45 s. Retinal fovea thickness, volume, and retinal nerve fibre layer (RNFL) thickness were measured preoperatively, and postoperatively at 1 h and 1 day, following FS-LASIK surgery. OCT (Optovue Inc., Fremont, CA, USA) performed 360° circular scans with a diameter of 3.45 mm centred on the optic disk.

Only the right eyes of the participants were included to be observed.

### Statistical Analysis

Volume and thickness of the inner retina (macula fovea, parafovea, and perifovea), and the thickness of the RNFL (superior, inferior, nasal, and temporal) were recorded pre- and postoperatively. The mean and standard deviation were calculated for both pre- and postoperative measurements. A paired *t*-test was utilised to determine any statistical difference between pre- and postoperative measurements. A *p*-value < 0.05 was considered statistically significant.

## Result

Eighteen males and nineteen females (*n* = 37) were enrolled in this study. Mean patient age was 22.94 ± 4.22 years. The thickness and volume of the macula declined significantly 1 h post operation (Table 1). By Day 1, post operation, five out of the six measures of the macula showed decreases compared to the preoperative values, but only four (fovea thickness and volume, parafovea thickness, and volume) of these were statistically significant (Table 1). All showed increases compared to immediate postoperatively.

**Table 1:** Mean macular fovea thickness and volume before and after femtosecond laser laser-assisted *in situ* keratomileusis (FS-LASIK) surgery.

	**Fovea thickness (μm)**	**Fovea volume (mm^**2**^)**	**Parafovea thickness (μm)**	**Parafovea volume (mm^**2**^)**	**Perifovea thickness (μm)**	**Perifovea volume (mm^**2**^)**
**Mean macular fovea thickness and volume (*****n*** **=** **37) (Mean** **±** **SD)**						
Before LASIK	67.18 ± 10.25	0.527 ± 0.081	124.29 ± 7.61	0.780 ± 0.047	98.83 ± 2.57	2.128 ± 0.199
1 h post-op	57.81 ± 10.5	0.454 ± 0.082	100.43 ± 15.98	0.634 ± 0.098	91.81 ± 1.48	1.941 ± 0.183
*t*-value	6.171	6.032	9.837	9.700	2.532	4.393
*P*-value	0.000*	0.000*	0.000*	0.000*	0.016*	0.000*
1 day post-op	62.24 ± 10.64	0.495 ± 0.088	120.18 ± 8.33	0.755 ± 0.052	100.75 ± 4.41	2.107 ± 0.187
*t*-value	3.620	3.220	2.901	2.910	0.694	0.525
*P*-value	0.001*	0.003*	0.006*	0.006*	0.492	0.603

The mean RNFL decreased 1 h postoperatively in the temporal and superior RNFL (Table 2). At 1 day post-operation, the RNFL was only significantly increased in the inferior portion compared to the preoperative measures. All measurements of macula and RNFL thickness and volume increased between 1 h postoperatively and 1 day postoperatively.

**Table 2:** Mean retinal nerve fibre layer thickness before and after FS-LASIK surgery.

	**Temporal**	**Superior**	**Nasal**	**Inferior**
**Mean retinal nerve fibre layer (*****n*** **=** **37) (μm) (Mean** **±** **SD)**				
Before LASIK	98.08 ± 10.14	129.24 ± 12.79	58.00 ± 9.08	124.29 ± 13.56
1 h post-op	86.86 ± 13.56	123.97 ± 17.16	58.67 ± 11.52	122.05 ± 17.42
*t*-value	4.926	2.265	0.443	0.997
*P*-value	0.000*	0.011*	0.66	0.325
1 day post-op	99.59 ± 12.4	126.78 ± 14.69	59.51 ± 9.98	129.24 ± 14.17
*t*-value	0.905	1.166	1.727	3.632
*P*-value	0.372	0.251	0.093	0.001*

## Discussion

Our study showed significant differences in preoperative and postoperative FS-LASIKs measurements of the macula and RNFL thickness as determined by OCT. Eight of ten measurements 1 h after FS-LASIKs were significantly different, whilst only five of ten measurements 1 day after FS-LASIKs were significantly different compared to preoperative measurements of the macula and RNFL. All measurements showed improvement between 1 and 1 day postoperatively. We hypothesise that our results are due to alterations in corneal architecture, thereby inducing artefactual OCT measurements.

There is conflicting evidence regarding measurements of macular edema and RNFL measurements by OCT in FS-LASIKs. Some studies have demonstrated changes within the macula and RNFL post FS-LASIKs (6, 8) and have attributed these effects to macular edema associated with IOP elevation during procedure. Elevation of IOP during LASIKs can be significant. In a previous study, intraoperative microkeratome LASIKs lead to IOP values of > 150 mmHg (9). Whilst FS-LASIKs leads to reduced IOP values during the suction, they lead to a greater period of maintenance of high IOP pressure, and there is uncertainty in comparative effect of FS-LASIKs on retinal measurements (10).

A range of microkeratome LASIK studies has demonstrated no detrimental effects on RNFL and macular thickness (3–5) and suggested that changes demonstrated *via* OCT are secondary to corneal aberrations, leading to artefactual measurements of the retina and fovea since there was no possibility that substantive decrease and the following increase of retina thickness can happen. The most likely cause of this phenomenon would be the reflectivity change, following the refractive procedure. Furthermore, larger studies with longer follow-up times have demonstrated that LASIKs is rarely associated with vitreoretinal pathology (11, 12).

Optical coherence tomography evaluates the reflectivity of posterior segment structures. Utilising these data, the RNFL thickness can consequently be calculated. FS-LASIK produces corneal spherical aberrations (13) and alters the refractive properties of the cornea. Consequently, this can interfere with OCT measurements, reducing reliability within the immediate postoperative period. These changes in refraction can be compensated for such as in scanning laser polarimetry (SLP) (14, 15). We should be aware of this measurement changes following the refractive procedure, since the myopia patients are the well-known potential high-risk group of glaucoma and retina disease in which OCT is one of the most important diagnostic tools.

## Conclusion

In our study, FS-LASIKs induced alterations in the inner retina and RNFL measurements by OCT. This is unlikely to be actual structural changes but is associated with changes in refractive properties of the cornea.

## Data Availability Statement

The raw data supporting the conclusions of this article will be made available by the authors, without undue reservation.

## Author Contributions

All authors listed have made a substantial, direct, and intellectual contribution to the work and approved it for publication.

## Funding

This work was supported by The Non-profit Central Research Institute Fund of Chinese Academy of Medical Sciences (2018PT32029).

## Conflict of Interest

The authors declare that the research was conducted in the absence of any commercial or financial relationships that could be construed as a potential conflict of interest.

## Publisher's Note

All claims expressed in this article are solely those of the authors and do not necessarily represent those of their affiliated organizations, or those of the publisher, the editors and the reviewers. Any product that may be evaluated in this article, or claim that may be made by its manufacturer, is not guaranteed or endorsed by the publisher.
